# A brain network for deep brain stimulation induced cognitive decline in Parkinson’s disease

**DOI:** 10.1093/brain/awac012

**Published:** 2022-01-17

**Authors:** Martin M. Reich, Joey Hsu, Michael Ferguson, Frederic L. W. V. J. Schaper, Juho Joutsa, Jonas Roothans, Robert C. Nickl, Anneke Frankemolle-Gilbert, Jay Alberts, Jens Volkmann, Michael D. Fox

**Affiliations:** 1 Berenson-Allen Center for Noninvasive Brain Stimulation and Division of Cognitive Neurology, Department of Neurology, Beth Israel Deaconess Medical Center, Harvard Medical School, Boston, MA, USA; 2 Harvard Medical School, Boston, MA, USA; 3 Department of Neurology, University Hospital and Julius-Maximilians-University, Wuerzburg, Germany; 4 School of Medicine, University of Pittsburgh, Pittsburgh, PA, USA; 5 Center for Brain Circuit Therapeutics, Department of Neurology, Psychiatry, and Radiology, Brigham & Women’s Hospital, Boston, MA, USA; 6 Turku Brain and Mind Center, Clinical Neurosciences, University of Turku, Turku, Finland; 7 Turku PET Centre, Neurocenter, Turku University Hospital, Turku, Finland; 8 Department of Neurosurgery, University Hospital and Julius-Maximilians-University, Wuerzburg, Germany; 9 Department of Biomedical Engineering, Duke University, Durham, NC, USA; 10 Department of Biomedical Engineering, Cleveland Clinic, Cleveland, OH, USA; 11 Martinos Center for Biomedical Imaging and Department of Neurology, Massachusetts General Hospital, Harvard Medical School, Boston, MA, USA

**Keywords:** Parkinson’s disease, deep brain stimulation, cognitive decline, connectivity

## Abstract

Deep brain stimulation is an effective treatment for Parkinson’s disease but can be complicated by side-effects such as cognitive decline. There is often a delay before this side-effect is apparent and the mechanism is unknown, making it difficult to identify patients at risk or select appropriate deep brain stimulation settings. Here, we test whether connectivity between the stimulation site and other brain regions is associated with cognitive decline following deep brain stimulation. First, we studied a unique patient cohort with cognitive decline following subthalamic deep brain stimulation for Parkinson’s disease (*n* = 10) where reprogramming relieved the side-effect without loss of motor benefit. Using resting state functional connectivity data from a large normative cohort (*n* = 1000), we computed connectivity between each stimulation site and the subiculum, an *a priori* brain region functionally connected to brain lesions causing memory impairment. Connectivity between deep brain stimulation sites and this same subiculum region was significantly associated with deep brain stimulation induced cognitive decline (*P* < 0.02).

We next performed a data-driven analysis to identify connections most associated with deep brain stimulation induced cognitive decline. Deep brain stimulation sites causing cognitive decline (versus those that did not) were more connected to the anterior cingulate, caudate nucleus, hippocampus, and cognitive regions of the cerebellum (*P*_FWE_ < 0.05). The spatial topography of this deep brain stimulation-based circuit for cognitive decline aligned with an *a priori* lesion-based circuit for memory impairment (*P* = 0.017). To begin translating these results into a clinical tool that might be used for deep brain stimulation programming, we generated a ‘heat map’ in which the intensity of each voxel reflects the connectivity to our cognitive decline circuit. We then validated this heat map using an independent dataset of Parkinson’s disease patients in which cognitive performance was measured following subthalamic deep brain stimulation (*n* = 33). Intersection of deep brain stimulation sites with our heat map was correlated with changes in the Mattis dementia rating scale 1 year after lead implantation (*r* = 0.39; *P* = 0.028).

Finally, to illustrate how this heat map might be used in clinical practice, we present a case that was flagged as ‘high risk’ for cognitive decline based on intersection of the patient’s deep brain stimulation site with our heat map. This patient had indeed experienced cognitive decline and our heat map was used to select alternative deep brain stimulation parameters. At 14 days follow-up the patient’s cognition improved without loss of motor benefit. These results lend insight into the mechanism of deep brain stimulation induced cognitive decline and suggest that connectivity-based heat maps may help identify patients at risk and who might benefit from deep brain stimulation reprogramming.

## Teaser

See Kuhn *et al.* (https://doi.org/10.1093/brain/awac101) for a scientific commentary on this article.

Reich *et al.* show that DBS-induced cognitive decline is associated with connectivity between the stimulation site and a specific brain network implicated in lesion-induced memory impairment. Transforming this network into a heat map can help identify DBS patients at risk of delayed-onset side-effects and guide reprogramming efforts.

## Introduction

Deep brain stimulation (DBS) to the subthalamic nucleus improves motor symptoms of Parkinson’s disease by 40–60% and improves quality of life.^1,2^ However, side-effects such as cognitive decline can occur in up to 15–20% of patients and dramatically limit this benefit.^3^ Cognitive decline can stem from neuronal damage during the surgery^3,4^ or from the effects of stimulation after the surgery.^5^ Stimulation-induced cognitive decline can go unrecognized because it can appear slowly over time and with DBS settings that work well for the primary motor symptoms.^5,6^ If recognized, cognitive decline may be reversible by changing the DBS settings.^5^ However, there is currently no effective way to predict who will develop stimulation-induced cognitive decline or select DBS settings to avoid this side effect.^3^

Accumulating evidence suggests that both the benefits and side-effects of DBS may come from stimulation of specific brain circuits.^7–10^ Normative human brain connectomes, based on specialized MRI scans from thousands of healthy individuals,^11^ can be used to identify these brain circuits without requiring connectivity data from the DBS patients themselves. Thus, this connectome approach can be applied to almost any clinical DBS dataset.^10,12,13^ Although this connectome approach has not yet been used to study post-DBS cognitive decline, it has been used to study post-stroke cognitive decline.^14^ Specifically, lesion locations causing memory impairment show stronger connectivity to the subiculum and a network of other memory-related brain regions.^14^ Given evidence that lesions and DBS sites that cause similar symptoms are connected to the same circuit,^15^ our lesion-based memory circuit provides an *a priori* template for studying DBS induced cognitive decline.

Here, we test whether connectivity between the stimulation site and other brain regions can predict cognitive decline following subthalamic nucleus (STN) DBS. We studied a unique Parkinson’s disease patient cohort with stimulation-induced cognitive decline following DBS (*n* = 10) where DBS reprogramming relieved the side-effect without loss of motor benefit.^5^ First, we used resting state functional connectivity data from a large cohort of healthy subjects (*n* = 1000) to test whether stimulation sites causing cognitive decline were more connected to an *a priori* region in the subiculum previously derived from brain lesions.^14^ Second, we performed a data-driven analysis to identify connections most associated with DBS induced cognitive decline and tested for alignment with connections most associated with post-stroke cognitive impairment. Accordingly, different cognitive metrics were used in post-stroke (episodic memory)^14^ and post-DBS cognitive decline (*n*-back task).^5^ Third, we generated a ‘heat map’ in which the intensity of each voxel reflects the connectivity to our cognitive decline circuit, which could provide a clinically useful template to predict the risk of cognitive decline post DBS and guide DBS programming. Finally, we validated this heat map by testing whether it could identify stimulation sites associated with cognitive decline in an independent dataset of Parkinson’s disease patients post STN DBS (*n* = 33).

## Materials and methods

### Overall study design and participants

Two independent cohorts of Parkinson’s disease patients with STN DBS were included in this study, a ‘DBS reprogramming’ cohort (*n* = 10) with two stimulation settings (one with and one without the cognitive side effects) per patient, and a ‘DBS validation’ cohort (*n* = 33) with one stimulation setting per patient. The DBS reprogramming cohort was used to test our *a priori* hypotheses, derive a data-driven circuit for DBS cognitive decline, and derive a heat map for DBS induced cognitive decline. The DBS validation cohort was used to test whether our heat map (derived from the reprogramming cohort) could identify patients at risk of cognitive decline in an independent dataset and potentially guide reprogramming.

The study was carried out in accordance with the Declaration of Helsinki and approved by the institutional review board of the Beth Israel Deaconess Medical Center, Boston, USA (IRB Protocol no. 2018P000128).

### DBS reprogramming cohort

The DBS reprogramming cohort consisted of 10 patients with Parkinson’s disease who experienced cognitive decline following STN-DBS measured with the *n*-back task.^5^ These patients then underwent DBS reprogramming which improved the cognitive side-effects [2-back condition, *F*(4,18) = 2.9945, *P* = 0.033, *n*^2^ = 0.247] without significant change in motor benefit [Unified Parkinson’s disease Rating Scale (UPDRS)-III: 30.0 ± 2.4 points versus 30.5 ± 5.6 points; *P* = 0.76]. The *n*-back task required the participant to repeat the *n*th item back (e.g. 0-back, 1-back and 2-back) in a sequentially presented list of items.^16^Additional details regarding this DBS cohort are available in the original publication.^5^ To our knowledge, this DBS cohort is unique in that each patient has two stimulation settings that differ only in the degree to which they caused cognitive side effects. As such, this was an ideal dataset in which to test our hypotheses and derive a DBS cognitive -decline circuit.

### Identification of stimulation sites: reprogramming cohort

As described previously,^5^ each subject’s electrode position was identified using a patient-specific DBS computer model generated using Cicerone v1.2.^5,17^ Briefly, co-registration between the frame image (T_1_-weighted) and a postoperative CT verified the intended surgical placement of the DBS electrode (Medtronic Electrode Model 3387 or 3389) and obtained the final stereotactic coordinates. This was displayed within the model system and volume of tissue activated (VTA) was computed for each subject before and after reprogramming using the built-in models of Cicerone v1.2.^18^ Images were then normalized into ICBM 2009b NLIN asymmetric space based on the local atlas registrations using a self-made MATLAB tool. To avoid any potential for bias in electrode localization or VTA modelling, the VTAs used for our analyses are identical to those used in the initial publication of this dataset.^5^

### Relating side-effects to stimulation site location: reprogramming cohort

To determine if side-effects could be predicted by VTA location alone, we performed a voxelwise paired *t*-test contrasting the location of the side effect VTAs with the reprogrammed VTAs, similar to prior work.^19^ Significance was assessed by permuting the data (switching the assignment of side effect versus reprogrammed VTA) and repeating the analysis 5000 times. The analysis was conducted using FSL PALM. We controlled for multiple comparisons using threshold free cluster-enhancement^20^ and family-wise error (FWE)-corrected α of 0.05. We also looked for significant voxels at a liberal uncorrected α of 0.05.

### Relating side-effects to stimulation site connectivity: reprogramming cohort

To compute connectivity between stimulation sites and other brain regions, we used a database of normative human connectome data as in prior work from our group.^10,21^ This resting state functional MRI blood oxygen level-dependent (BOLD) dataset was obtained from 1000 healthy subjects using a 3T Siemens scanner as part of the Brain Genomics Superstruct Project.^11,22^ Preprocessing of the BOLD data included regression of global signal, white matter and CSF signals, and the six motion parameters, as well as spatial smoothing with a 6 mm full width at half maximum kernel as previously described.^11^ The resolution of the connectome data and calculated connectivity maps was 2 × 2 × 2 mm.

First, we computed connectivity between each stimulation site and an *a priori* region of interest at the junction of the hippocampus and retrosplenial cortex (subiculum-retrosplenial continuum).^14^ This region of interest came from a prior study showing that connectivity between lesion locations and this region of interest was sensitive and specific for memory impairment.^14^ We tested the hypothesis that connectivity between DBS sites and this same region of interest would be associated with post-DBS memory impairment. A paired *t*-test was performed on the difference in VTA connectivity to this region of interest (side effect versus reprogrammed VTAs), and α level of 0.05 was selected for statistical significance.

Second, we used a hypothesis-free data-driven approach to identify connections associated with DBS induced cognitive decline. The approach was similar to prior work,^10,23^ but modified to compare VTA connectivity within a subject rather than across subjects. Whole-brain functional connectivity maps were generated for the side-effect-causing and reprogramming VTAs for each subject as follows. First, the mean BOLD time course was extracted from voxels within each VTA and correlated with the time courses of all other brain voxels. This procedure was performed using the resting-state fMRI data for each subject in our 1000-subject connectome dataset, generating 1000 ‘r’-maps for each VTA. Then, the Fisher z-transformation was applied to each ‘r’-map, and the resulting ‘Fz’-maps were averaged to generate the whole-brain connectivity map for each VTA. A statistical group ‘cognitive decline map’ was then obtained from these connectivity maps via a permutation-based, voxel-wise paired *t*-test, performed within FSL PALM and using threshold free cluster-enhancement^20^ and 5000 permutations. FWE correction was applied and an α level of 0.05 was selected to identify voxels demonstrating significant preferential positive connectivity to the side-effect-causing VTAs. Note that this was the same statistical analysis we used to contrast the VTA locations.

Third, we tested whether our data-driven map of DBS-induced cognitive decline was similar to our published map of lesion-induced memory impairment.^14^ This map of lesion-induced memory impairment consists of voxels significantly more connected to lesions causing amnesia than lesions causing other symptoms.^14^ We used spatial correlation to quantify the similarity between the DBS- and lesion-derived maps and then recalculated this metric based on permuted versions of the DBS dataset (swapping side-effect-causing and reprogramming VTAs) to test whether the observed value was stronger than expected by chance (10 000 permutations and an α level of 0.05), similar to a recent work from our group.^15^

Finally, we tested whether our DBS-induced cognitive decline network was distinct from our previously published network for DBS-induced motor improvement.^10^ First, we compared the topography of the two networks using the same spatial correlation and permutation technique detailed above. Second, we computed the connectivity between each VTA (clinical and reprogramming) and each network (cognitive decline and motor improvement). We hypothesized that connectivity of VTAs to our cognitive decline network would be associated with *n*-back task performance, but not motor improvement (measured as change in UPDRS-III scores with DBS on versus DBS off, both assessed after overnight medication OFF).^5^ Conversely, we hypothesized that connectivity to our motor improvement network would be associated with motor improvement, but not *n*-back task performance.

### Local network heat map projection: reprogramming cohort

We transformed our data-driven map of connections associated with DBS induced cognitive decline into a ‘heat map’ that could more easily be used to guide DBS programming. This heat map could allow for the evaluation of new VTAs through their direct overlap on the heat map—a much less computationally intensive process compared to recalculating the connectivity of each VTA with our cognitive decline network. The heat map was generated by calculating the spatial correlation between the whole-brain connectivity map for each brain voxel and the ‘reference’ cognitive decline map. The spatial correlation values were then Fisher *z*-transformed. Thus, the value at each voxel reflects the connectivity of that voxel to our cognitive decline map. In theory, the average value of heat map voxels encompassed by a VTA should reflect the risk of cognitive decline symptoms resulting from that VTA.

### Heat map validation in independent cohort

The DBS validation cohort consisted of 33 patients with Parkinson’s disease who consecutive received STN-DBS in Wurzberg, Germany between 2011 and 2015. This was a subset of patients from our prior study^10^ who completed cognitive testing (baseline and 1-year follow-up Mattis Dementia Rating scale, MDRS). Since passage of the lead through the head of the caudate during DBS surgery has been associated with cognitive decline,^3^ all lead trajectories were blindly evaluated in Elements (Brainlab Inc.) by a neurosurgeon (R.C.N.) trained in stereotactic surgery, and any subjects meeting this criterion were also excluded (*n* = 1). The clinical details are presented in Table 1.

**Table 1:** Demographic characteristics and chronic stimulation parameters of the independent cohort subdivided in patients with clinically relevant cognitive decline (≥5 points reduction in the 1-year follow-up) and control subjects

Characteristic	Validation cohort (*n* = 33)	
	Cognitive decline	Control
*n*	6	27
Age at surgery, years	59.2 (±7.1)	60.7 (±8.0)
Disease duration prior to surgery, years	11.7 (±2.7)	12.9 (±4.5)
Baseline motor severity (UPDRS-III)	50.2 (±16.9)	50.0 (±10.8)
Baseline side-effect severity (MDRS)	141.5 (±2.0)	141.1 (±2.5)
Follow-up time, months	12.0	12.0
Motor improvement over baseline, %	42.8 (±11.3)	55.5 (±15.9)
Follow-up side effect severity (MDRS)	131.8 (±4.4)	140.2 (±2.6)
Stimulation parameters		
Amplitude, mA	3.2 (±1.4)	3.2 (±0.8)
Pulse width, µs	60	60
Frequency, Hz	150.0 (±27.4)	146.7 (±23.9)

Values are presented as mean (±SD).

DBS leads in the validation cohort were localized as described in the original publication of this dataset.^10^ Briefly, postoperative images were linearly co-registered to preoperative MRI using SPM12 (http://www.fil.ion.ucl.ac.uk/spm/software/spm12/; postoperative MRI). Images were then normalized into ICBM 2009b NLIN asymmetric space using the SyN approach implemented in advanced normalization tools (http://stnava.github.io/ANTs/) based on the preoperative MRI. DBS electrode contacts were localized within MNI space and VTAs were simulated using Lead-DBS software.^10^ As with our reprogramming cohort, we used previously published VTAs to avoid any potential bias in lead localization or VTA generation.

We computed the intersection between the VTAs from each patient in our validation cohort (*n* = 33) with the heat map generated using the data from our reprogramming cohort. The average value of all heat map voxels intersected by each VTA was calculated to generate a single ‘cognitive decline risk score’ for each subject. We then tested for a correlation between this risk score and measured cognitive decline (change between baseline and 1-year follow-up MDRS). We also stratified patients into three risk groups based on the intersection of their VTAs with our heat map. Specifically, we split patients into three equal sized groups for low (−0.1 ± 0.11, *n* = 11), medium (0.09 ± 0.04, *n* = 10) and high risk (0.234 ± 0.08, *n* = 11). ANOVA were used to analyse variance between these three groups and *posthoc* we tested if two groups differ in cognitive changes between baseline and 1-year follow-up.

For the patients in our high-risk group, we tested whether we could find new stimulation that would lead to a lower risk of cognitive decline. This was done by simulating monopolar VTAs at all four contacts, for the left and right electrodes, using a single standard VTA (3.2 mA; 60 µs). Parameters for this single standard VTA were based on the average stimulation settings from our independent reprogramming cohort (Table 1). In total, there were 16 stimulation settings tested for each patient (four contacts on both the left and right electrode). For each setting, we computed a cognitive risk score based on intersection with our heat map. One patient in our high-risk group returned for routine clinical follow-up during the time our analyses were being done. As the patient had experienced cognitive decline, the DBS settings were changed based on our heat map. A UPDRS score and MDRS score were repeated at 14 days.

### Statistical analysis

All statistical analyses were performed with GraphPad Prism 6 (Version 2015) and an α level of 0.05 was selected as significant. For voxel-wise analyses, which were performed with FSL PALM as described above, FWE correction was applied with α level of 0.05.

### Data availability

The data that support the findings of this study are available on request from the corresponding author. The data are not publicly available due to their containing information that could compromise the privacy of research participants.

## Results

### DBS reprogramming of cognitive decline: stimulation location and connectivity profile

**Figure 1:**
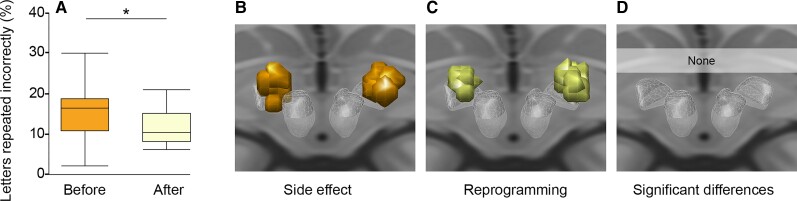
**Cognitive decline following STN-DBS can be improved with reprogramming, but there is no significant difference in the stimulation location.** DBS reprogramming resulted in significant improvement in working memory performance in a previously published cohort of 10 Parkinson’s disease patients. (**A**) Reproduced from Frankemolle *et al*.^5^). VTAs associated with impaired working memory (**B**) and the reprogrammed VTAs associated with improved working memory (**C**) showed no significant difference in stimulation location (**D**). *P < 0.05.

**Figure 2:**
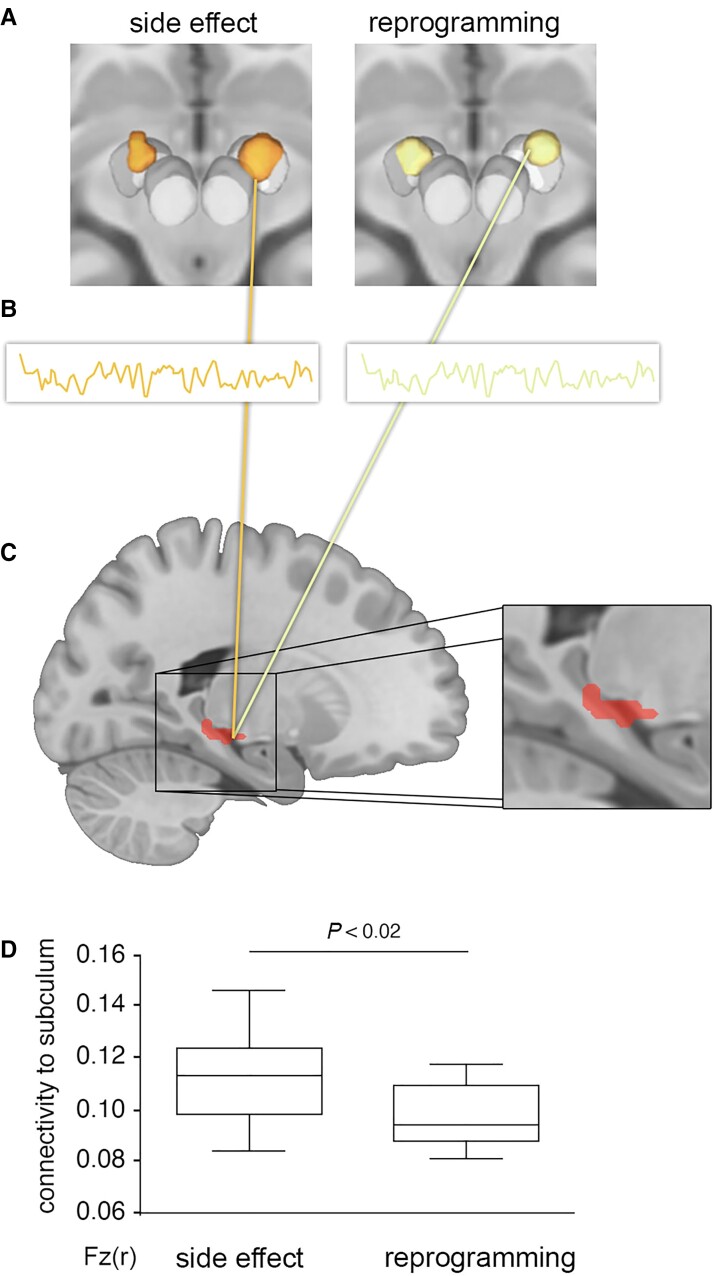
**Connectivity of stimulation site to the subiculum is associated with cognitive decline**. For each patient, the VTA associated with cognitive decline (**A**, *left*) and the reprogrammed VTA associated with improvement (**A**, *right*) were used as seed regions for a functional connectivity analysis, leveraging a normative connectome dataset from 1000 healthy subjects (**B**). We computed connectivity between each VTA and an *a priori* region of interest in the subiculum previously linked to lesion-induced memory impairment (**C**, red, reproduced from Ferguson *et al*.^14^). VTAs associated with cognitive decline were significantly more connected to the subiculum than reprogrammed VTAs associated with improvement (**D**).

In our DBS re-programming cohort,^5^ comparison between the location of VTAs associated with the cognitive decline side effect and the location of VTAs after reprogramming failed to identify any voxels significantly associated with cognitive decline, either before or after multiple comparisons correction (Fig. 1). In contrast, functional connectivity between these same VTAs and our *a priori* region of interest in the subiculum was significantly associated with cognitive decline (Fig. 2). Consistent with our hypothesis and prior lesion-based results,^14^ VTAs causing cognitive decline were more connected to the subiculum than reprogrammed VTAs that improved this decline (*P* = 0.015).

**Figure 3:**
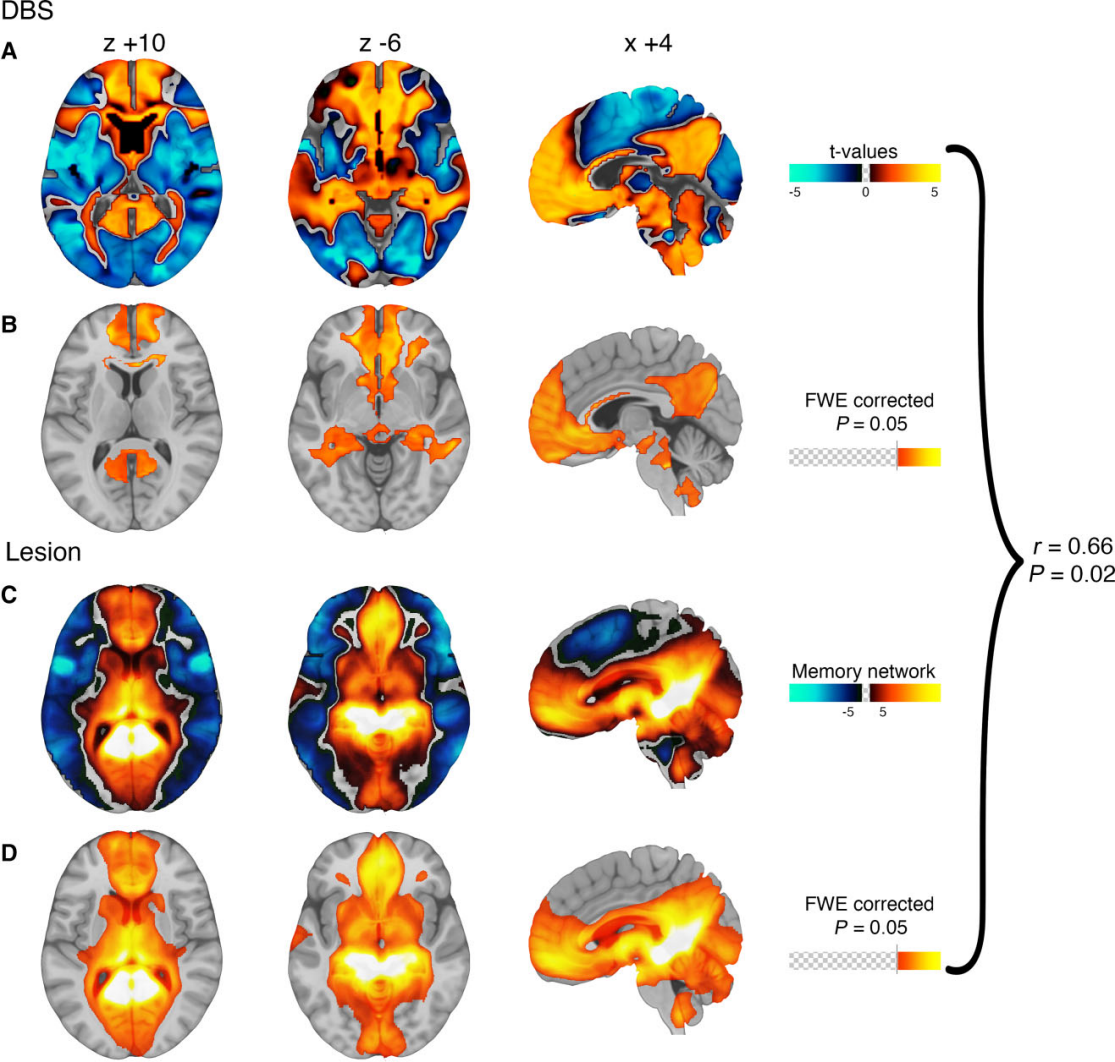
**Connections associated with DBS-induced cognitive decline are similar to connections associated with lesion-induced memory impairment**. Connections associated with DBS induced cognitive decline are shown using no threshold (**A**) and after FWE correction for multiple comparisons (**B**). Connections associated with lesion-induced memory impairment are shown using no threshold (**C**) and after FWE correction for multiple comparisons (**D**) as previously reported in Ferguson *et al*.^14^ Warm colours denote voxels more connected to VTAs/lesions associated with cognitive impairment while cool colours denote voxels more connected to reprogrammed VTAs/lesions not associated with cognitive impairment. The unthresholded maps (**A** and **C**) can be compared using spatial correlation and permutation analysis and are significantly similar to one another (*r* = 0.66; *P* = 0.017). The FWE corrected maps (**B** and **D**) show that similar voxels are statistically significant across both analyses.

Using a data-driven analysis, VTAs causing cognitive decline (versus reprogrammed VTAs) showed stronger functional connectivity to the bilateral anterior cingulate, caudate nucleus, hippocampus, and cognitive regions of the cerebellum (FWE-corrected *P* < 0.05, Fig. 3 and Table 2). The topography of this DBS-based cognitive decline circuit aligned well with our *a priori* lesion-based circuit for memory impairment (Fig. 3).^14^ These circuits were significantly more similar than expected by chance (spatial *r* = 0.66; *P* = 0.017, permutation test). In contrast, the topography of our DBS-based cognitive decline network to our previously published DBS-based motor improvement network showed a very low spatial correlation (spatial *r* = 0.29, *P* = 0.52; Supplementary Fig. 1).^10^ As such, only 9% of the spatial variance aligns between the two networks, with 91% of the variance being unique to each network.

**Table 2:** Brain areas (coordinates of regions and peak-voxel in MNI space) with significantly increased connectivity (FWE corrected)

*P* (FWE corr.)	*k*	Region	Coordinates (mm)			*t* value
			*x*	*y*	*z*	
**Cognitive decline**						
0.05	26292	Frontal orbital cortex	28	12	−20	4.85
			−12	24	−18	4.68
			−26	14	−22	4.54
		Subcallosal cortex	0	28	−2	4.44
		Caudate nucleus	−16	−6	26	4.37
			−12	14	−4	4.10
		Anterior cingulate cortex	4	34	−2	4.32
		Posterior cingulate cortex	16	−42	28	4.28
			−20	−64	24	3.92
		Angular gyrus	44	−46	24	4.15
			40	−60	−38	3.84
		Hippocampus	32	−30	−8	3.85
0.05	1439	Cerebellum, left crus I	−40	−58	−36	4.06
0.05	811	Cerebellum, left IX	−4	−52	−58	4.01

Further supporting a differentiation between networks, we found that connectivity of VTAs to our cognitive decline network was correlated with baseline *n*-back task performance (*r* = 0.663 *P* = 0.013) but not motor improvement (*r* = 0.31 *P* = 0.1914). Conversely, connectivity of VTAs to our motor improvement network was correlated with UPDRS-III improvement (*r* = 0.597, *P* = 0.034) but not *n*-back task performance (*r* = 0.083 *P* = 0.41). With reprogramming, the change in connectivity to our cognitive decline network was significantly greater than the change in connectivity to our motor improvement network (−0.1007 ± 0.08801 versus −0.034 ± 0.02883; *P* = 0.0195), consistent with a change in *n*-back scores but no change in UPDRS.^5^ Finally, the improvement in the *n*-back task with reprogramming was correlated with the change in connectivity to the cognitive decline network (R = −0.61; *P* = 0.029) but not correlated with change in connectivity to the motor improvement network (R = 0.109; *P* = 0.38).

### Network heat maps and individual optimized neuromodulation

**Figure 4:**
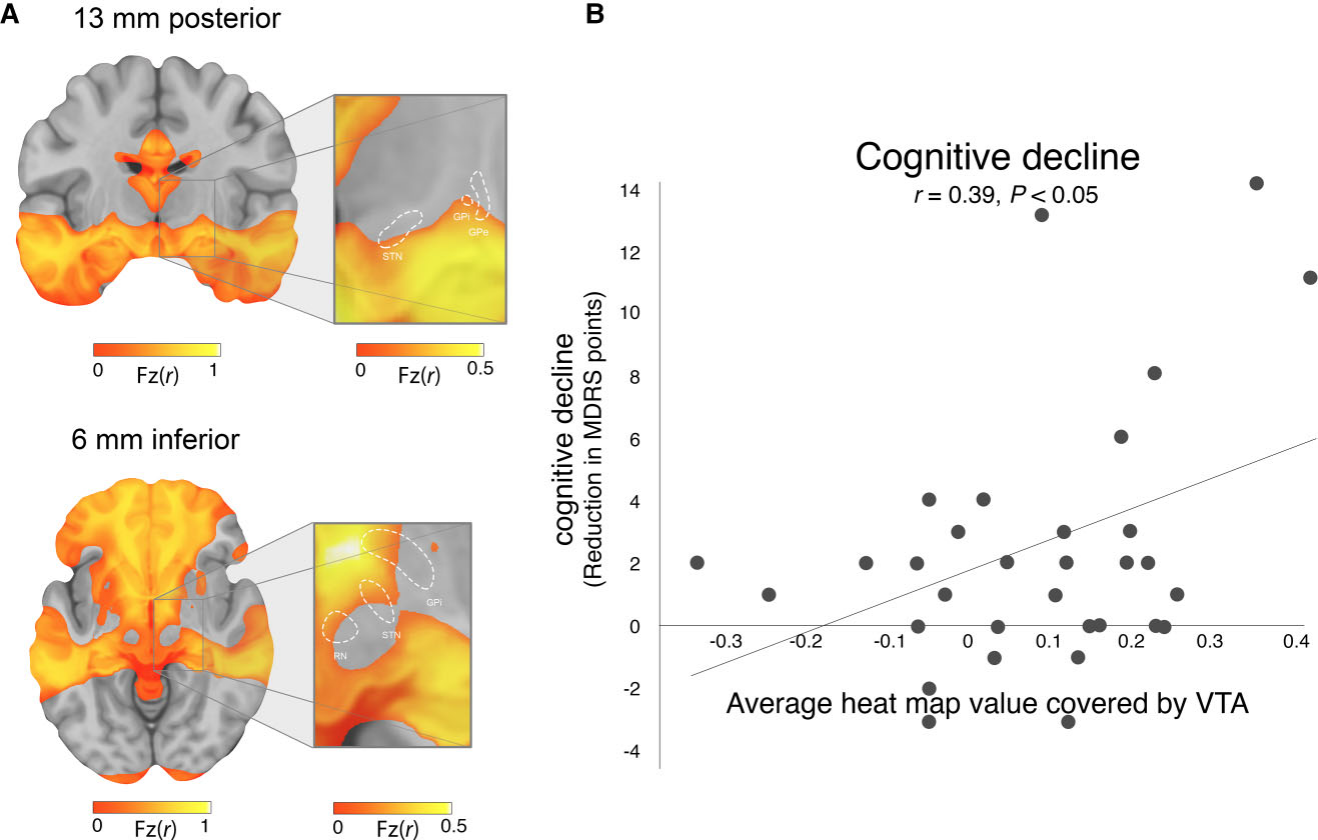
**Cognitive decline heat map and validation in an independent cohort**. To begin translating our network results into a clinical tool, we generated a heat map where the intensity at each voxels reflects the connectivity of that voxel to our cognitive decline network (**A**). In the area of the STN (white outline), there is an anterior-posterior and dorsal-ventral gradient. We then tested this heat map in an independent cohort of Parkinson’s disease patients with STN DBS and MDRS at baseline and 1 year after DBS (**B**). Intersection between VTAs and our cognitive decline heat map was corelated with cognitive decline measured at 1 year (*r* = 0.39; *P* < 0.05).

After transforming our DBS-based cognitive decline circuit into a voxel-wise heat map, we observed that voxels in the ventral anterior STN showed stronger connectivity to our cognitive decline circuit than voxels in the dorsal posterior STN (Fig. 4). Using an independent STN DBS validation dataset (*n* = 33), we found that intersection of VTAs with our cognitive decline heat map correlated with cognitive decline measured at 1 year post-DBS (change in MDRS (*r* = 0.39, *P* < 0.05, Fig. 4).

**Figure 5:**
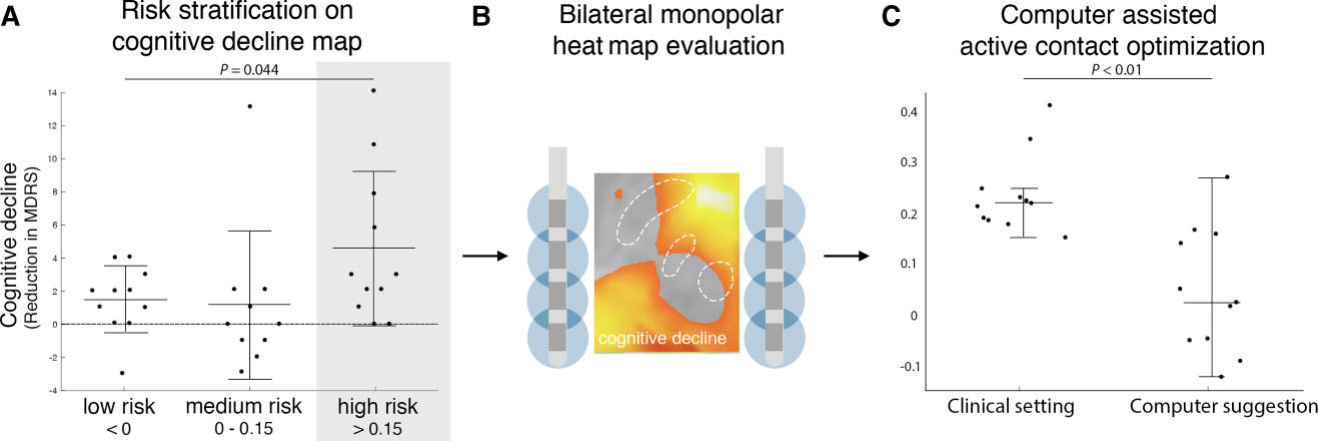
**Using the cognitive decline heat map for risk assessment and potential reprogramming**. Patients were stratified into three risk groups based on VTA overlap with our cognitive decline heat map (**A**). Parkinson’s disease patients in the high-risk cohort showed significantly more cognitive decline than patients in low-risk cohort and medium risk cohort. We simulated all possible monopolar electrode choices for patients in the high-risk group (**B**) and choose the setting that would minimize the risk of cognitive decline (**C**). In 8 of 11 patients there were alternative programming settings where cognitive decline could potentially be reduced. One of these high-risk patients presented for routine clinical follow-up and was reprogrammed, moving from a high risk setting to a medium-risk setting based on the heat map (**D**). The MDRS improved by 7 points, while parkinsonism was unchanged (48% to 52% UPDRS III reduced to baseline).

When patients in our validation cohort were stratified into risk groups based on the degree of VTA overlap with the cognitive decline heat map, there was a significant difference in cognitive decline across groups (one-way ANOVA: *P* = 0.0448; Fig. 5). Parkinson’s disease patients in our high-risk group showed significantly more cognitive decline than patients in our medium risk cohort (−4.5 ± 4.6 points versus −1.1 ± 4.5 points on MDRS, *P* = 0.010; Fig. 5A) or low risk cohort (−4.5 ± 4.6 points versus −1.0 ± 1.9 points on MDRS, *P* = 0.029; Fig. 5A).

**Figure 6:**
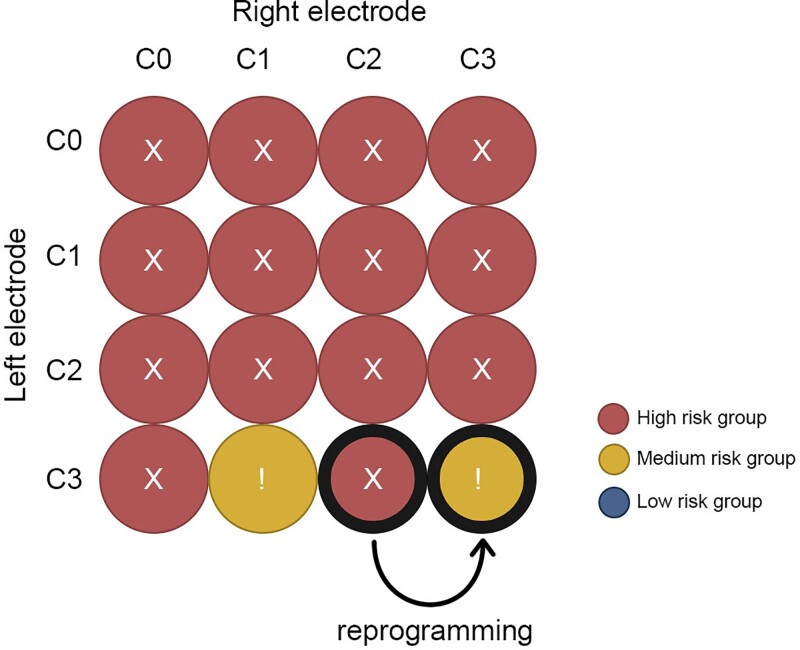
**DBS programming prediction for an individualized treatment** An exhaustive evaluation of this patients on the heat map of cognitive decline detected a preferable electrode contact combination to reduce the risk of cognitive side-effects. We called back this patient and changed stimulation settings from right lead C2 to C3. Parkinsonism was equally controlled (48–52% UPDRS III reduced to baseline) 14 days in the medication OFF state, while the MDRS improved by 7 points to previous DBS settings (still 6 points reduced to preoperative baseline).

Using a set of simulated VTAs covering all possible monopolar electrode choices for each patient, we found that patients in our high-risk group could potentially be changed to an alternative setting that would decrease their risk of cognitive decline (*P* < 0.01; Wilcoxon rank sum test). Specifically, 8/11 patients (72%) in the high risk cohort had an alternative DBS setting that would place them in the lower risk group, predicting a significant benefit in terms of cognitive decline (clinical setting: 0.22 ± 0.08 versus ‘low risk’ setting 0.03 ± 0.12; *P* < 0.01; Fig. 5C). Results for one example patient are shown (Fig. 6) who was seen back in clinic for DBS reprogramming. His DBS site was changed from the clinical setting to an alternative setting with a hypothesized lower risk of cognitive decline based on our heat map. Cognition improved from −11 points on the MDRS (preoperative baseline versus 1 year post DBS) to −6 points (preoperative baseline versus 14 days after reprogramming). Parkinson’s disease motor symptom control by STN DBS was unchanged (52% improvement in UPDRS III versus 48% improvement after reprogramming measured in medication OFF).

## Discussion

In this study, there are four main findings. First, cognitive decline following STN DBS for Parkinson’s disease is associated with connectivity between the stimulation site and a specific network of other brain regions. Second, this network aligns with regions previously implicated in lesion-induced memory impairment.^14^ Third, this network can be converted into a voxel-wise heat map and intersection of VTAs with this heat map is correlated with cognitive decline in an independent dataset. Finally, we illustrate how this heat map might be used to predict the risk of DBS induced cognitive decline and guide reprogramming. These results have implications for understanding the mechanism of DBS induced cognitive decline and for avoiding this side effect in DBS patients.

### Connectivity profiles of DBS effects

Our results align well with a growing literature suggesting that connectivity between DBS sites and brain networks is responsible for DBS induced effects.^10,24^ Further, our results provide further support that a normative connectome can be used to identify these networks. While there is value in obtaining connectivity data from the patients themselves^19,24^ or using advanced imaging to directly measure remote DBS effects^25,26^ this is often not possible in clinical settings. In such cases, using a normative connectome averaged across thousands of subjects can serve as useful approximation of the connectivity in each patient.^10^ While this approach may sacrifice subject-specific differences in connectivity, this limitation is largely outweighed by the ability to produce robust and reproducible connectivity estimates. Connectomes specific to the population of interest can also be used (e.g. from Parkinson’s disease patients) but are generally of lower quality (and quantity) than normative connectomes and appear to have little impact on results.^10,27^ This normative connectome approach has worked robustly across brain lesions,^28^ noninvasive brain stimulation,^27^ and DBS.^29^ This approach requires no specialized imaging of the patient, only a record of the stimulation location based on routine clinical scans, and thus can be broadly applied in research or clinical settings.

The current study pushes the boundary of this connectivity-based approach and provides an important advance. Specifically, the normative connectome was used to estimate connectivity differences between stimulation settings within the same patient, rather than connectivity difference across patients. These within-subject VTAs were highly overlapping and appeared nearly identical, both visually and statistically (Fig. 1). Furthermore, the cohort size was remarkably small (*n* = 10). In spite of this, statistically significant and reproducible connectivity differences could be identified. This suggests that a within-subject analysis in a small but well-defined cohort may have the power to detect connectivity differences associated with specific clinical effects that would typically require a much larger cohort when performing a more traditional cross-subject analysis. The increased power of a within-subject design may come from the fact that this design controls for many factors that introduce confounds into cross-subject analyses such as genetics, electrode trajectory, or disease progression. Further, this dataset was unique in that there was no change in motor benefit between stimulation settings, allowing one to relate connectivity differences to cognitive decline.^5^

### Alignment between DBS effects and lesion networks

An important finding is that our brain network for DBS induced cognitive decline aligned almost optimally with a recently published network for lesion-induced memory impairment^14^ and is different to the network of DBS induced motor benefits^10^ (Supplementary Fig. 1). The concordance of the cognitive network occurred despite that fact that different cognitive metrics were used in the two studies (*n*-back task versus episodic memory). However, this concordance is consistent with prior work suggesting that the lesion-based network for memory impairment generalizes across metrics for measuring memory impairment.^14^ The concordance is also consistent with a growing literature suggesting that lesion locations and DBS sites that cause similar effects converge on common brain networks.^15,30–33^ As such, brain networks derived from brain lesions may be valuable in guiding future DBS trials or avoiding other DBS induced side effects. High frequency DBS (40–180 Hz) is thought to have a functional effect on brain circuits that is similar to lesions, possibly through altering network ‘communication’ via information overload.^34^ As such, one would expect that high frequency DBS sites that impair a function would be connected to the same brain circuit as lesions that impair that function. This is exactly what we observed here for memory/cognitive decline and what we have observed previously for DBS sites and lesions associated with depression.^15^ However, it is possible that different relationships could be observed for alternate DBS settings that act differently than a lesion. For example, low frequency DBS may drive or activate a circuit and could have the opposite functional effect from high frequency DBS or lesions.^35^ Future work is required to determine whether DBS sites connected to our memory circuit, but using alternative DBS parameters, could improve memory or cognition rather than inhibit it.^36^

### Converting connectivity profiles into heat maps

One barrier to translating connectome results into better DBS programming is the relatively long computational time that would be required to re-compute connectivity with each change in the stimulation site or DBS parameters. One cannot simply look at the connectivity network for cognitive decline (e.g. Fig. 3) and know which DBS settings should be used to avoid this network. By converting a connectivity profile into a heat map, one can easily test whether a stimulation site intersects this heat map, choose a site that avoids this heat map, or perform an exhaustive review of a patient’s stimulation settings to select an ‘ideal’ setting within a matter of seconds. An advantage of the heat map approach is that the connectivity information has all been ‘precalculated’, allowing a clinician to quickly assess the risk of cognitive decline from any given stimulation site based on simple intersection between the stimulation site and the heat map. This heat map can be distributed as a single small file and this calculation can be performed in less than a second. In contrast, computing connectivity between the stimulation site and our cognitive decline network (without the heat map) requires access to our 1000-subject connectome, specialized software, and computational resources that are not routinely accessible to DBS clinicians. This conversion of a connectivity network into a heat map has been used for OCD^37^ but to our knowledge this is the first use and validation of this approach for a DBS side effect.

Our cognitive decline heat map, derived using a within-subject analysis in a small cohort (*n* = 10), was correlated with cognitive decline across subjects in an independent cohort. This is an important advantage, as small within-subject studies can be easily conducted in a research setting (e.g. *n* = 10 patients), then applied more broadly to predict DBS effects across different clinical populations. The ability to predict (side) effects in any patient, based only on the location of the patient’s electrodes and their DBS settings, greatly broadens the impact and clinical utility of this work. Furthermore, our heat map could be used to improve imaging-based implantation strategies and provide the neurosurgeon with data regarding risk of delayed side effects that may not be apparent based on microelectrode recordings or acute effects of intra-operative test stimulation. So far, our cognitive decline heat map applies only to STN DBS for Parkinson’s disease. Whether this heat map is relevant for cognitive outcomes following STN DBS for other conditions, such as obsessive compulsive disorder, requires future study.^38^

### Clinical implications on programming DBS

Titration of stimulation parameters is a critical component of DBS therapy and focuses predominantly on acute motor benefit and acute side-effects evident in real time.^29,39^ Delayed side effects such as cognitive decline are therefore problematic and can go unrecognized for years or be attributed to progression of the disease itself rather than DBS.^3^ Even when DBS is suspected, it is often unclear which DBS settings should be changed to relieve the side effect. Previous studies have suggested reprogramming to more dorsal contacts can help relive cognitive decline.^3,5,40^ Such observations are consistent with our heat map, which showed a clear dorsal ventral gradient. However, our heat map also showed a clear anterior posterior gradient and depending on the exact location of an individual’s electrode, simply moving to a more dorsal contact may not avoid our cognitive decline network. Further work is needed to determine whether reprogramming based on our cognitive decline heat map can outperform other heuristics.

### Limitations

Different cognitive metrics were used to define cognitive decline across datasets (working memory vs episodic memory versus MDRS). However, this should bias us against the present findings of convergence across datasets. Second, the discovery dataset used to generate our DBS induced cognitive decline network was remarkably small (*n* = 10). We could not have used a dataset this small to derive a cognitive decline network based on difference across subjects. Using a unique within subject analysis, this small dataset was sufficient to generate significant findings that were then validated in independent datasets. Third all of our primary analyses were retrospective. Whether our cognitive decline network or heat map can predict cognitive decline remains to be tested prospectively. Finally, our single patient that was reprogrammed should be taken as an illustration of how this approach might be used in a clinical reprogramming scenario. The clinical value of this approach must be assessed using a randomized and blinded clinical trial.

## Conclusion

Our study shows that DBS-induced cognitive decline is associated with connectivity between the stimulation site and a specific brain network previously implicated in lesion-induced memory impairment. Transforming this network into a heat map may help identify DBS patients at risk of delayed-onset side-effects and guide reprogramming efforts.

## Supplementary Material

awac012_Supplementary_Data

## Abbreviations

DBS: deep brain stimulation
MDRS: Mattis Dementia Rating Scale
STN: subthalamic nucleus
UPDRS: Unified Parkinson’s disease Rating Scale
VTA: volume of tissue activated
