# Supplementary material for: A brain network for deep brain stimulation induced cognitive decline in Parkinson’s disease
Source: Brain. 2022 Jan 17;145(4):1410–21. doi: 10.1093/brain/awac012 (PMC9129093; doi:10.1093/brain/awac012)
Supplement: awac012_Supplementary_Data [file awac012_supplementary_data.pdf]

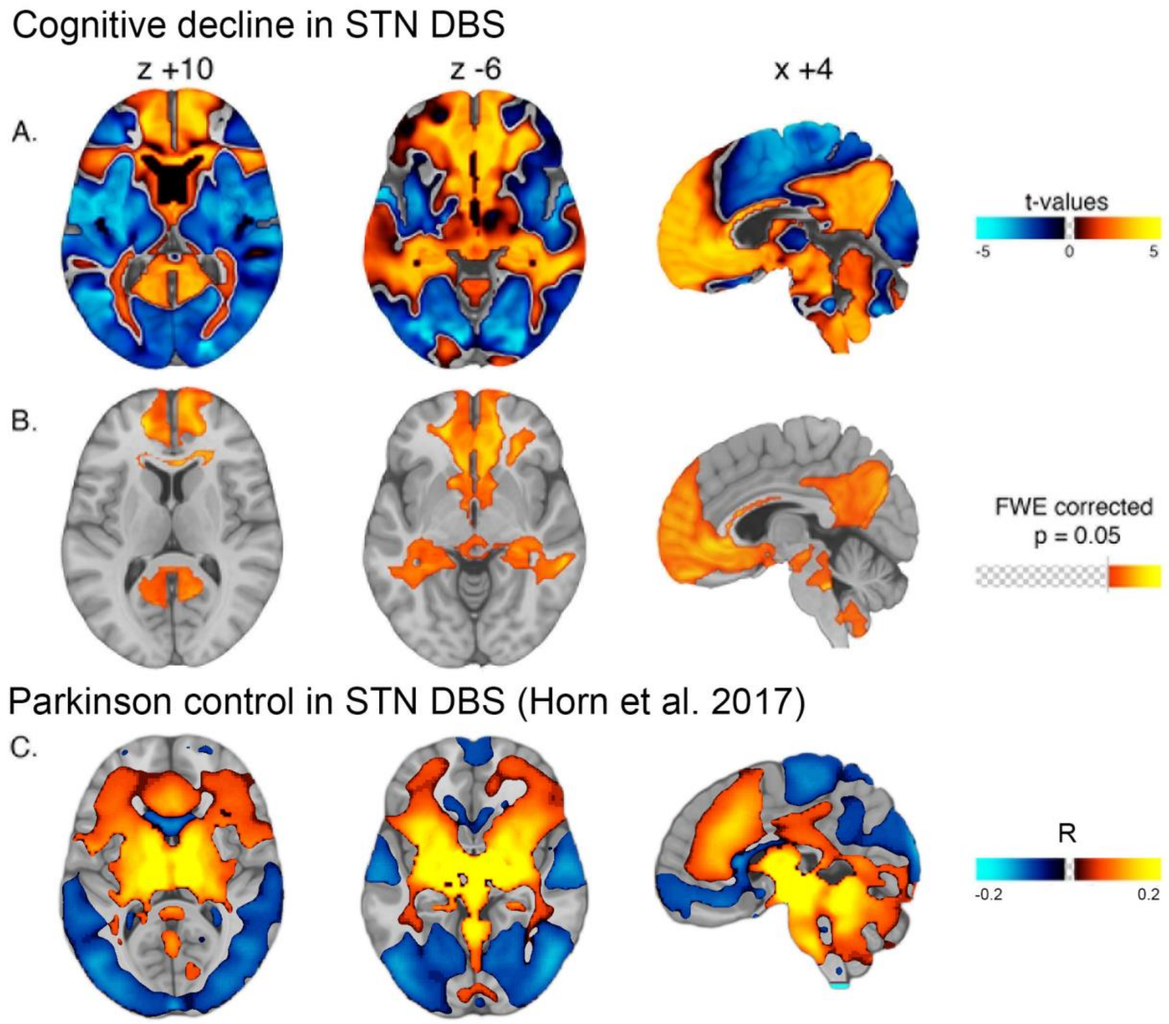

***<u>Figure S1</u>*:** **Topography of DBS-based cognitive decline network to previously published DBS-based motor improvement network** [10].

Both networks showed a very low spatial correlation (r = 0.29, p = 0.52 Pearson correlation). We computed the connectivity between our simulated VTAs to the motor improvement network and cognitive decline network and showed that correlation to the network is associated with clinical performance (UPDRS-III to motor improvement network: r = 0.597, p = 0.034; n-back task to cognitive decline network: r = 0.663 p = 0.013). Results were (side-) effect specific, as our cognitive decline map showed no predictive utility for UPDRS-III (r = 0.31 p = 0.1914) and our motor improvement network showed no predictive utility for cognitive decline (r = 0.083 p = 0.41).

10. Horn A, Reich M, Vorwerk J, et al. Connectivity Predicts deep brain stimulation outcome in Parkinson disease. *Ann Neurol*. Jul 2017;82(1):67-78. doi:10.1002/ana.24974
